# Supplementary material for: Multi‐dataset identification of innovative feature genes and molecular mechanisms in keratoconus
Source: J Cell Mol Med. 2024 Sep 19;28(18):e70079. doi: 10.1111/jcmm.70079 (PMC11412914; doi:10.1111/jcmm.70079)
Supplement: Supplementary file 2 — Material S2. [file JCMM-28-e70079-s002.zip › 7.GSEA analysis/4.MYRF/KEGG_WNT_SIGNALING_PATHWAY.html]

Details for gene set KEGG\_WNT\_SIGNALING\_PATHWAY[GSEA]

|  || Dataset | MYRF.group.cls#h\_versus\_l |
| Phenotype | group.cls#h\_versus\_l |
| Upregulated in class | l |
| GeneSet | KEGG\_WNT\_SIGNALING\_PATHWAY |
| Enrichment Score (ES) | -0.34236625 |
| Normalized Enrichment Score (NES) | -1.4590901 |
| Nominal p-value | 0.029166667 |
| FDR q-value | 0.21707988 |
| FWER p-Value | 0.815 |
Table: GSEA Results Summary

  

Fig 1: Enrichment plot: KEGG\_WNT\_SIGNALING\_PATHWAY      
 Profile of the Running ES Score & Positions of GeneSet Members on the Rank Ordered List

  

| SYMBOL | TITLE | RANK IN GENE LIST | RANK METRIC SCORE | RUNNING ES | CORE ENRICHMENT || 1 | SFRP1 | na | 79 | 0.886 | 0.0408 | No |
| 2 | CAMK2B | na | 283 | 0.659 | 0.0671 | No |
| 3 | NFATC2 | na | 1281 | 0.423 | 0.0588 | No |
| 4 | PPP3R2 | na | 2013 | 0.356 | 0.0550 | No |
| 5 | RAC3 | na | 2468 | 0.325 | 0.0577 | No |
| 6 | CHP2 | na | 3253 | 0.283 | 0.0488 | No |
| 7 | CXXC4 | na | 3527 | 0.270 | 0.0540 | No |
| 8 | CAMK2A | na | 4292 | 0.245 | 0.0438 | No |
| 9 | WNT10B | na | 5576 | 0.206 | 0.0167 | No |
| 10 | SFRP2 | na | 6410 | 0.180 | 0.0014 | No |
| 11 | DKK4 | na | 6791 | 0.170 | -0.0013 | No |
| 12 | APC2 | na | 8076 | 0.137 | -0.0317 | No |
| 13 | MAPK9 | na | 8409 | 0.129 | -0.0350 | No |
| 14 | WNT16 | na | 8449 | 0.128 | -0.0299 | No |
| 15 | ROCK1 | na | 8751 | 0.121 | -0.0327 | No |
| 16 | CSNK1A1 | na | 8757 | 0.121 | -0.0270 | No |
| 17 | FZD1 | na | 9008 | 0.116 | -0.0286 | No |
| 18 | WNT5A | na | 9048 | 0.115 | -0.0241 | No |
| 19 | PLCB3 | na | 9511 | 0.106 | -0.0323 | No |
| 20 | CREBBP | na | 9688 | 0.102 | -0.0325 | No |
| 21 | PLCB2 | na | 9762 | 0.100 | -0.0297 | No |
| 22 | LRP6 | na | 9917 | 0.097 | -0.0294 | No |
| 23 | FZD3 | na | 10112 | 0.094 | -0.0304 | No |
| 24 | CSNK2A1 | na | 10382 | 0.089 | -0.0339 | No |
| 25 | CAMK2G | na | 10986 | 0.078 | -0.0475 | No |
| 26 | PSEN1 | na | 11022 | 0.078 | -0.0447 | No |
| 27 | VANGL1 | na | 11319 | 0.073 | -0.0497 | No |
| 28 | PRKX | na | 11663 | 0.068 | -0.0563 | No |
| 29 | FRAT2 | na | 11738 | 0.067 | -0.0552 | No |
| 30 | PPP2R5A | na | 11827 | 0.066 | -0.0545 | No |
| 31 | FZD5 | na | 11924 | 0.065 | -0.0542 | No |
| 32 | MAPK10 | na | 12440 | 0.057 | -0.0663 | No |
| 33 | WNT3 | na | 12453 | 0.057 | -0.0638 | No |
| 34 | TP53 | na | 12977 | 0.051 | -0.0765 | No |
| 35 | TBL1Y | na | 13017 | 0.050 | -0.0752 | No |
| 36 | SMAD4 | na | 13356 | 0.045 | -0.0828 | No |
| 37 | FZD4 | na | 13390 | 0.045 | -0.0815 | No |
| 38 | CHD8 | na | 13469 | 0.044 | -0.0816 | No |
| 39 | WNT2 | na | 13551 | 0.043 | -0.0819 | No |
| 40 | TBL1XR1 | na | 13771 | 0.041 | -0.0862 | No |
| 41 | ROCK2 | na | 13790 | 0.040 | -0.0848 | No |
| 42 | PRKCA | na | 14202 | 0.036 | -0.0949 | No |
| 43 | TBL1X | na | 14433 | 0.033 | -0.1000 | No |
| 44 | CTBP2 | na | 14461 | 0.033 | -0.0991 | No |
| 45 | CCND1 | na | 14657 | 0.031 | -0.1033 | No |
| 46 | RBX1 | na | 14789 | 0.030 | -0.1056 | No |
| 47 | CTBP1 | na | 14847 | 0.029 | -0.1058 | No |
| 48 | PPP2R1B | na | 15086 | 0.027 | -0.1114 | No |
| 49 | TCF7L2 | na | 15138 | 0.027 | -0.1116 | No |
| 50 | LRP5 | na | 15153 | 0.026 | -0.1107 | No |
| 51 | SFRP5 | na | 15211 | 0.026 | -0.1111 | No |
| 52 | PORCN | na | 15272 | 0.025 | -0.1116 | No |
| 53 | SFRP4 | na | 15437 | 0.023 | -0.1152 | No |
| 54 | FZD9 | na | 15491 | 0.023 | -0.1156 | No |
| 55 | CTNNB1 | na | 15600 | 0.022 | -0.1177 | No |
| 56 | FRAT1 | na | 15761 | 0.020 | -0.1213 | No |
| 57 | PRKCG | na | 15973 | 0.018 | -0.1266 | No |
| 58 | NLK | na | 16181 | 0.016 | -0.1318 | No |
| 59 | FBXW11 | na | 16395 | 0.014 | -0.1373 | No |
| 60 | JUN | na | 16671 | 0.011 | -0.1447 | No |
| 61 | BTRC | na | 16745 | 0.011 | -0.1463 | No |
| 62 | RAC2 | na | 17259 | 0.006 | -0.1608 | No |
| 63 | SKP1 | na | 17295 | 0.006 | -0.1615 | No |
| 64 | PLCB4 | na | 17554 | 0.004 | -0.1688 | No |
| 65 | CTNNBIP1 | na | 17579 | 0.003 | -0.1693 | No |
| 66 | SMAD3 | na | 17747 | 0.002 | -0.1741 | No |
| 67 | PPP3R1 | na | 17799 | 0.001 | -0.1755 | No |
| 68 | WNT8A | na | 17911 | 0.000 | -0.1787 | No |
| 69 | DAAM2 | na | 18075 | -0.001 | -0.1833 | No |
| 70 | SMAD2 | na | 18091 | -0.001 | -0.1837 | No |
| 71 | PPP2R5D | na | 18454 | -0.005 | -0.1939 | No |
| 72 | PPARD | na | 18532 | -0.006 | -0.1959 | No |
| 73 | MAPK8 | na | 18612 | -0.006 | -0.1979 | No |
| 74 | RHOA | na | 18827 | -0.008 | -0.2036 | No |
| 75 | CHP1 | na | 19165 | -0.012 | -0.2128 | No |
| 76 | GSK3B | na | 19466 | -0.016 | -0.2207 | No |
| 77 | FZD6 | na | 19569 | -0.017 | -0.2228 | No |
| 78 | PRKACA | na | 19584 | -0.017 | -0.2224 | No |
| 79 | PPP2R5C | na | 19773 | -0.019 | -0.2269 | No |
| 80 | PPP2R5E | na | 19812 | -0.020 | -0.2271 | No |
| 81 | CSNK2B | na | 19992 | -0.022 | -0.2312 | No |
| 82 | FZD8 | na | 20046 | -0.022 | -0.2316 | No |
| 83 | CSNK2A2 | na | 20546 | -0.028 | -0.2447 | No |
| 84 | WNT4 | na | 20821 | -0.031 | -0.2511 | No |
| 85 | WNT3A | na | 20924 | -0.033 | -0.2525 | No |
| 86 | NFATC3 | na | 21139 | -0.035 | -0.2569 | No |
| 87 | PRICKLE1 | na | 21151 | -0.035 | -0.2555 | No |
| 88 | WNT9A | na | 21202 | -0.036 | -0.2552 | No |
| 89 | CUL1 | na | 21468 | -0.039 | -0.2610 | No |
| 90 | DAAM1 | na | 21505 | -0.040 | -0.2601 | No |
| 91 | RAC1 | na | 21936 | -0.045 | -0.2704 | No |
| 92 | PPP3CA | na | 22219 | -0.049 | -0.2761 | No |
| 93 | DVL1 | na | 22373 | -0.050 | -0.2781 | No |
| 94 | WIF1 | na | 22535 | -0.053 | -0.2802 | No |
| 95 | PPP2R1A | na | 22646 | -0.054 | -0.2808 | No |
| 96 | CSNK1A1L | na | 22742 | -0.056 | -0.2808 | No |
| 97 | PPP3CB | na | 22763 | -0.056 | -0.2786 | No |
| 98 | CACYBP | na | 22982 | -0.059 | -0.2821 | No |
| 99 | DVL3 | na | 23247 | -0.063 | -0.2866 | No |
| 100 | AXIN1 | na | 23328 | -0.065 | -0.2858 | No |
| 101 | SENP2 | na | 23426 | -0.066 | -0.2854 | No |
| 102 | CCND3 | na | 23579 | -0.069 | -0.2864 | No |
| 103 | RUVBL1 | na | 24302 | -0.081 | -0.3034 | No |
| 104 | PPP2R5B | na | 24593 | -0.086 | -0.3076 | No |
| 105 | SIAH1 | na | 24671 | -0.088 | -0.3056 | No |
| 106 | MAP3K7 | na | 24724 | -0.088 | -0.3028 | No |
| 107 | CER1 | na | 25675 | -0.106 | -0.3251 | No |
| 108 | PRKCB | na | 25770 | -0.108 | -0.3225 | No |
| 109 | CCND2 | na | 25792 | -0.109 | -0.3178 | No |
| 110 | PPP2CB | na | 25840 | -0.109 | -0.3139 | No |
| 111 | PPP2CA | na | 26826 | -0.131 | -0.3360 | Yes |
| 112 | WNT1 | na | 27000 | -0.135 | -0.3344 | Yes |
| 113 | NFAT5 | na | 27258 | -0.141 | -0.3350 | Yes |
| 114 | EP300 | na | 27396 | -0.145 | -0.3319 | Yes |
| 115 | TCF7L1 | na | 27666 | -0.152 | -0.3323 | Yes |
| 116 | MYC | na | 27707 | -0.153 | -0.3260 | Yes |
| 117 | MMP7 | na | 27871 | -0.157 | -0.3231 | Yes |
| 118 | WNT10A | na | 28144 | -0.164 | -0.3229 | Yes |
| 119 | DVL2 | na | 28187 | -0.166 | -0.3161 | Yes |
| 120 | PPP3CC | na | 28351 | -0.171 | -0.3124 | Yes |
| 121 | APC | na | 28648 | -0.180 | -0.3122 | Yes |
| 122 | VANGL2 | na | 28844 | -0.186 | -0.3088 | Yes |
| 123 | WNT2B | na | 28930 | -0.189 | -0.3021 | Yes |
| 124 | WNT7B | na | 29404 | -0.203 | -0.3059 | Yes |
| 125 | TCF7 | na | 30107 | -0.227 | -0.3151 | Yes |
| 126 | FZD10 | na | 30271 | -0.233 | -0.3085 | Yes |
| 127 | WNT8B | na | 30486 | -0.242 | -0.3029 | Yes |
| 128 | PLCB1 | na | 30796 | -0.254 | -0.2995 | Yes |
| 129 | NKD2 | na | 31352 | -0.283 | -0.3018 | Yes |
| 130 | PRKACG | na | 31385 | -0.285 | -0.2888 | Yes |
| 131 | NFATC1 | na | 31545 | -0.293 | -0.2792 | Yes |
| 132 | WNT9B | na | 31651 | -0.298 | -0.2677 | Yes |
| 133 | CAMK2D | na | 31910 | -0.312 | -0.2600 | Yes |
| 134 | WNT5B | na | 32169 | -0.328 | -0.2515 | Yes |
| 135 | WNT7A | na | 32275 | -0.335 | -0.2382 | Yes |
| 136 | FZD2 | na | 32361 | -0.340 | -0.2241 | Yes |
| 137 | SOX17 | na | 32510 | -0.351 | -0.2113 | Yes |
| 138 | AXIN2 | na | 32625 | -0.360 | -0.1971 | Yes |
| 139 | DKK2 | na | 32646 | -0.361 | -0.1801 | Yes |
| 140 | NFATC4 | na | 32887 | -0.379 | -0.1686 | Yes |
| 141 | WNT6 | na | 33111 | -0.398 | -0.1557 | Yes |
| 142 | DKK1 | na | 33390 | -0.430 | -0.1428 | Yes |
| 143 | LEF1 | na | 33408 | -0.432 | -0.1223 | Yes |
| 144 | PRKACB | na | 33438 | -0.435 | -0.1019 | Yes |
| 145 | PRICKLE2 | na | 33890 | -0.502 | -0.0905 | Yes |
| 146 | FOSL1 | na | 33933 | -0.510 | -0.0669 | Yes |
| 147 | FZD7 | na | 34203 | -0.571 | -0.0469 | Yes |
| 148 | NKD1 | na | 34268 | -0.591 | -0.0200 | Yes |
| 149 | WNT11 | na | 34512 | -0.683 | 0.0062 | Yes |
Table: GSEA details [plain text format]

  

Fig 2: KEGG\_WNT\_SIGNALING\_PATHWAY      
 Blue-Pink O' Gram in the Space of the Analyzed GeneSet

  

Fig 3: KEGG\_WNT\_SIGNALING\_PATHWAY: Random ES distribution      
 Gene set null distribution of ES for **KEGG\_WNT\_SIGNALING\_PATHWAY**
